# Characteristics of gene expression in frozen shoulder

**DOI:** 10.1186/s12891-022-05762-3

**Published:** 2022-08-25

**Authors:** Hiroaki Nishimoto, Shoji Fukuta, Naoshi Fukui, Koichi Sairyo, Tetsuo Yamaguchi

**Affiliations:** 1 Graduate School of Technology, Industrial and Social Sciences, Tokushima University, Minami-Jyosanjima 1-1, Tokushima, 770-8502 Japan; 2 Department of Orthopaedic Surgery, National Hospital Organization Kochi National Hospital, Kochi, Japan; 3 Graduate School of Arts and Sciences, The University of Tokyo, Tokyo, Japan; 4 Clinical Research Center, National Hospital Organization Sagamihara Hospital, Kanagawa, Japan; 5 Department of Orthopedics, Institute of Biomedical Sciences, Tokushima University Graduate School, Tokushima, Japan

**Keywords:** Frozen shoulder, Gene expression, Fibrosis, Chondrogenesis

## Abstract

**Background:**

Severe frozen shoulder (FS) is often resistant to treatment and can thus result in long-term functional impairment. However, its etiology remains unknown. We hypothesized that gene expression of FS would vary by synovial location.

**Methods:**

The synovial tissues of patients with FS were collected prospectively and analyzed for the expression of 19 genes. Synovial tissues from patients with rotator cuff tear (RCT) or shoulder instability (SI) were also analyzed as controls. A total of 10 samples were analyzed from each group. The specimens were arthroscopically taken from three different locations: rotator interval (RI), axillary recess (AX), and subacromial bursa (SAB). Total RNA was extracted from the collected tissues and was analyzed by real-time polymerase chain reaction for the following genes: matrix metalloproteinases (MMPs); tissue inhibitors of metalloproteinases (TIMPs); inflammatory cytokines (*IL1B, TNF,* and *IL6*); type I and II procollagen (*COL1A1* and *COL2A1*); growth factors (*IGF1* and *TGFB1*); neural factors (*NGF* and *NGFR*); *SOX9;* and *ACTA2*.

**Results:**

Site-specific analysis showed that *MMP13, IL-6, SOX9*, and *COL1A1* were increased in all three sites. Four genes (*MMP3*, *MMP9*, *COL2A1*, and *NGFR*) were increased in the AX, *MMP3* in the RI, and *NGFR* in the SAB were increased in the FS group than in the RCT and SI groups. In the FS group, there was a correlation between the expression of genes related to chondrogenesis (*MMP2*, *IGF1*, *SOX9*, *COL2A1*, *NGF*, and *NGFR*) or fibrosis (*MMP9*, *TGFB1*, and *COL1A1*).

**Conclusion:**

The expression levels of numerous *MMPs*, pro-inflammatory cytokines, and collagen-related genes were increased in the FS group, suggesting that catabolic and anabolic changes have simultaneously occurred. In addition, genes related to chondrogenesis or fibrosis were highly expressed in the FS group, which might have affected the range of motion limitation of the shoulder. Compared to RI and SAB, the AX was the most common site of increased expression in FS. Analyzing the lower region of the shoulder joint may lead to the elucidation of the pathogenesis of FS.

## Background

Frozen shoulder (FS) is a common shoulder disease that causes progressive loss of shoulder motion. It affects approximately 2–5% of the general population and is usually found in people aged 40–60 years [1]. FS, also known as adhesive capsulitis, is associated with a high incidence of synovial proliferation in the rotator interval (RI) and subacromial bursa (SAB) [1, 2]. In addition, it is also associated with the thickening of the joint capsule and formation of adhesions [1]. However, the cause of these remains unknown.

The underlying pathophysiologic processes of FS involve capsular inflammation with subsequent fibrosis. These processes are modulated by mediators, including inflammatory cytokines, growth factors, matrix metalloproteinases (MMPs), and tissue inhibitors of metalloproteinases (TIMPs) [2–4]. Bunker analyzed samples of the coracohumeral ligament and the RI capsule of patients with FS [5]. In these samples, there was active fibroblast proliferation and transformation to myofibroblasts, which are considered the main effector cells in joint fibrosis [5].　However, several studies reported that the number of myofibroblasts did not increase in FS [6, 7]. Hence, the role of myofibroblasts in FS remains unclear.

Nerve growth factor (NGF) is a widely recognized mediator of chronic pain; it has been reported to promote chondrogenic differentiation of mesenchymal stem cells and myofibroblast differentiation of the NIH/3T3 cell line [8, 9]. NGF exerts its action through two types of receptors: the high-affinity tyrosine kinase A receptor and the low-affinity NGF receptor (p75NGFR) [10]. p75NGFR was significantly increased in the synovial tissues of patients with FS, suggesting that p75NGFR potentiates the action of NGF [11].

Magnetic resonance imaging (MRI) in FS demonstrates changes in the thickening of the RI and axillary recess (AX) [12]. In molecular biology, Hagiwara et al. analyzed tissue samples from the RI, middle glenohumeral ligament (MGHL), and inferior glenohumeral ligament (IGHL) and reported that the expression levels of genes involved in chondrogenesis were increased in the MGHL and IGHL [6]. Lho et al. reported that inflammatory cytokines were expressed at significantly high levels in the SAB of patients with FS [2].

The aforementioned results of imaging and gene expression studies suggest that the pathogenesis of FS varies by site. This study aimed to characterize the gene expression of the affected tissues in patients with FS by collecting synovial tissue samples from three different locations (RI, AX, and SAB).

## Materials and methods

### Patients and tissue samples

This study included patients who received shoulder arthroscopic surgery of the shoulder over a 5-year period (2016–2020) at Kochi National Hospital, Tokushima University Hospital, or Taoka Hospital. We studied patients with primary FS who received arthroscopic capsular release. Patients with shoulder instability (SI) undergoing arthroscopic stabilization and patients with rotator cuff tear (RCT) undergoing rotator cuff repair were included as the control groups.

The criteria for inclusion were as follows: (1) severe night pain; (2) a restricted range of flexion or abduction that was less than 90°; (3) poor response to range of motion (ROM) exercises conducted for at least 2 months prior to surgery; and (4) symptom duration > 2 months. All patients with FS had normal rotator cuff, labrum, long head of the biceps, and acromioclavicular joint on plain radiograph and MRI. Patients with diabetes, cardiovascular, thyroid diseases, or cancer were excluded. The diagnosis of FS was confirmed through arthroscopy. The arthroscopic findings that were diagnostic of FS were hypervascular synovitis and thickened rotator interval and capsule. All patients of the frozen shoulder group were in the freezing (painful) and frozen (adhesive) phases because they had both severe pain and limited ROM. There were no patients in the thawing phase. The inclusion criteria for the SI group consisted of several episodes of dislocation and a Bankart or a Hill-Sachs lesion confirmed on arthroscopy. The inclusion criteria for the RCT group consisted of the absence of ROM limitation and the presence of full-thickness tears measuring ≦ 30 mm in width.

Synovial specimens (10–20 mg) used in this study were collected during arthroscopic shoulder capsular release, rotator cuff repair, or Bankart repair. The specimens were taken from the RI, AX, and SAB before using a radiofrequency device. None of the patients received intra-articular injections within three weeks prior to surgery. All samples were obtained after the participants gave their informed consent under protocols approved by our Institutional Review Board.

### Quantitative polymerase chain reaction

Human synovial tissue obtained at the time of surgery was immediately placed in RNAlater (Thermo Fisher, Carlsbad, CA, USA) and was stored at − 80 °C until processing. Total RNA was extracted using the RNeasy Mini Kit (Qiagen, Hilden, Germany) and TRIzol reagent (Thermo Fisher) following the manufacturer’s instructions. To generate complementary DNA (cDNA), 1 μg of total RNA was reverse transcribed using the Sensiscript RT Kit (Qiagen). cDNA samples were aliquoted and stored at − 80 °C.

Real time-PCR was performed using the GoTaq qPCR Master Mix (Promega, Madison, WI, USA) on a LineGene real-time thermal cycler (BioFlux, Tokyo, Japan). The settings of the PCR were as follows: 95° °C for 30 s, with 50 cycles performed at 95 °C for 15 s, 55 °C for 15 s, and 72 °C for 30 s. The PCR results of MMPs (*MMP1, MMP2, MMP3, MMP9,* and *MMP13*), TIMPs (*TIMP1, TIMP2,* and *TIMP3*), inflammatory cytokines (interleukin [*IL*] *1 B, IL6,* and tumor necrosis factor [*TNF*]), growth factors (insulin-like growth factor-1 [*IGF1*] and transforming growth factor-β [*TGFB1*]), neural factors (*NGF and NGFR*), type I and II procollagen (*COL1A1* and *COL2A1*), α-smooth muscle actin (*ACTA2*), and a transcriptional factor SRY-box transcription factor 9 (*SOX9*) were analyzed using the prepared cDNA as a template. Glyceraldehyde-3-phosphate dehydrogenase (*GAPDH*) was used as an internal control. We performed a log (base e) transformation of the normalized relative gene expression levels. The primer sequences used in the PCR analysis are shown in Table 1.

**Table 1:** Details of primer pairs used

Gene	Primer 1	Primer 2
MMP1	5'-CTG GCC ACA ACT GCC AAA TG-3'	5'-CTG TCC CTG AAC AGC CCA GTA CTT A-3'
MMP2	5'-TGT TTG TGC TGA AGG ACA CAC TAA-3'	5'-CTT GCG AGG GAA GAA GTT GTA GTT-3'
MMP3	5'-TCA TTT TGG CCA TCT CTT CC-3'	5'-TGG CTC CAG GAA TTT CTC T-3'
MMP9	5'-AAG GCG CAG ATG GTG GAT-3'	5'-GCA GGA TGT CAT AGG TCA CGT A-3'
MMP13	5'-TAA GGA GCA TGG CAC TTC T-3'	5'-GTC TGG CGT TTT TGG ATG TT-3
TIMP1	5'-GCT GGA AAA CTG CAG GAT GGACTC-3'	5'-CTG GAA GCC CTT TTC AGA GCC TTG-3'
TIMP2	5'-CTT AGT GTT CCC TCC CTC AAA GAC-3'	5'-AAG AAG TGA GTG TGT CAC CAA AGC-3'
TIMP3	5'-AGC TTC CGA GAG TCT CTG TG-3'	5'-GTA GCA GGA CTT GAT CTT GCA GTT-3'
IL1B	5'-TCG CCA GTG AAA TGA TGG CTT-3'	5'-GTC CAT GGC CAC AAC AAC TGA-3'
IL6	5'-ACT CAC CTC TTC AGA ACG AAT TG-3'	5'-CCA TCT TTG GAA GGT TCA GGT TG-3'
TNF	5'-CTC TCT CCC CTG GAA AGG AC-3'	5'-TCA CCC ATC CCA TCT CTC TC-3'
IGF1	5'-ACA TCT CCC ATC TCT CTG GAT TTC CTT TTG C-3’	5'-CCC TCT ACT TGC GTT CTT CAA ATG TAC TTC C-3'
TGFB1	5'-AGG ACC TCG GCT GGA AGT GGA T-3'	5'-AGG CGC CCG GGT TAT GCT-3'
COL1A1	5'-AGC CTG GGG CAA GAC ATG ATT-3'	5'-TTG CTT GTC TGT TTC CGG GTT G-3'
COL2A1	5'-AAT TCC TGG AGC CAA AGG AT-3'	5'-AGG ACC AGT TGC ACC TTG AG-3'
SOX9	5'-AGC GAA CGC ACA TCA AGA C-3'	5'-CTG TAG GCG ATC TGT TGG GG-3'
NGF	5'-GGC AGA CCC GCA ACA TTA CT-3'	5'-CAC CAC CGA CCT CGA AGT C-3'
NGFR	5'-TGG CTC CCC TCT ATT TAG CAT G-3'	5'-ATA CTT GCA AGC CCC CAA AC-3'
ACTA2	5'-GAG ATC TCA CTG ACT ACC TCA TGA-3'	5'-AGA GCT ACA TAA CAC AGT TTC TCC TTG A-3'

Abbreviations: *MMP*, matrix metalloprotease; *TIMP,* tissue inhibitor of metalloproteinase; *IL*, interleukin; *TNF*, tumor necrosis factor-α; *IGF1*, insulin-like growth factor-1; *TGFB1*, transforming growth factor- β; *COL1A1*, α 1 type I collagen; *COL2A1*, α 1 type II collagen; *SOX9*, SRY-box transcription factor 9; *NGF*, nerve growth factor; *NGFR*, nerve growth factor receptor; *ACTA2*, actin alpha 2

### Statistical analyses

Data were analyzed statistically using the *GraphPad* Prism 9 (*GraphPad* Software, San Diego, CA, USA). Statistically significant differences were determined by the Brown Forsythe and the Welch ANOVA test or the Kruskal–Wallis test with Dunns multiple comparison test. The correlations between gene expression levels were evaluated using Spearman′s correlation analysis. Statistical significance was set at *p* < 0.05.

## Results

### Patient demographics

The study included 30 patients (11 women, 19 men), 10 (3 women, 7 men) of whom had SI (mean age, 27.0 years; range, 16–44 years), 10 (5 women, 5 men) had FS (mean age, 58.5; range, 41–69 years), and the remaining 10 (3 women, 7 men) had RCT (mean age, 67.8 years; range, 56–74 years). The age, gender, and disease duration of FS patients are shown in Table 2. Patients with SI were significantly younger than patients with FS and RCT patients (*p* = 0.01 and *p* < 0.001, respectively). There was no significant difference in the body mass index among the three groups (SI; mean 22.7 [18.4–27.0], FS; mean 21.9 [17.9–26.0], and RCT; mean 22.9 [18.2–27.0]).

**Table 2:** Demographics of patients with frozen shoulder

No	Age	Sex	Symptom duration, mo
1	63	female	3
2	41	male	3
3	60	female	4
4	56	male	12
5	68	male	3
6	62	female	4
7	69	male	2
8	65	female	6
9	46	female	3
10	55	male	14

### Comparison of gene expression in the three groups

**Fig. 1:**
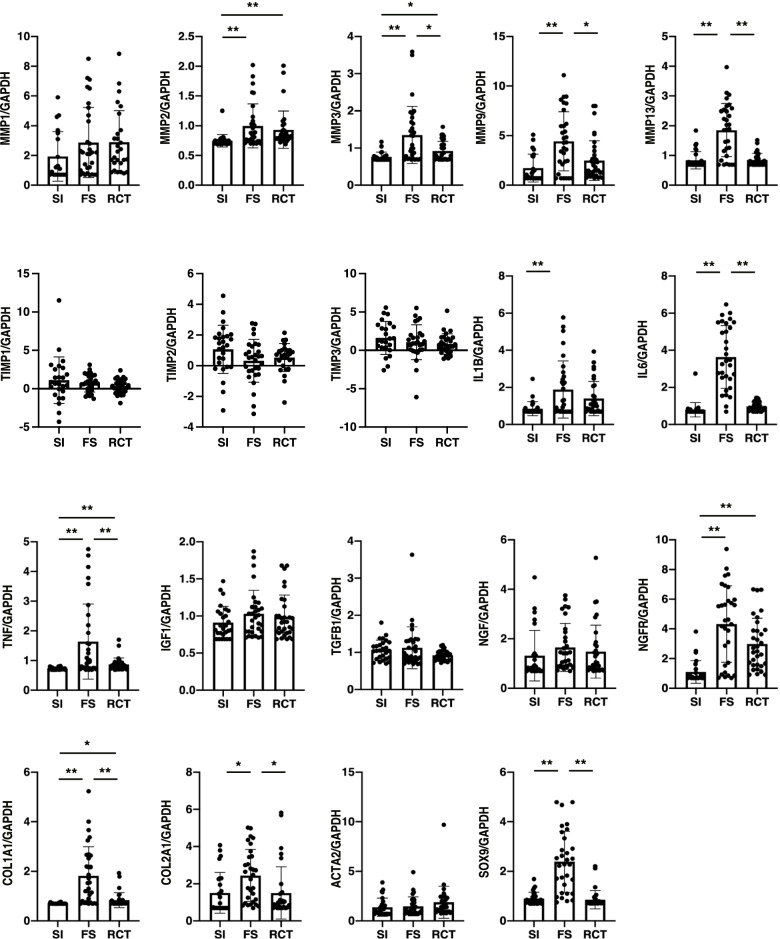
Expression levels of the genes in the synovium. mRNA expression levels of each gene are represented as a ratio of intensity of each RT-PCR gene band to that of the corresponding *GAPDH*. The mean values of the samples from the three sites were determined. *n* = 10 for each group. Data are presented on a logarithmic y scale. * *p* < 0.01; **p < 0.05; the Brown Forsythe and the Welch ANOVA test. Abbreviation: SI, shoulder instability; FS, frozen shoulder; RCT, rotator cuff tear; *MMP*, matrix metalloprotease; *TIMP*, tissue inhibitor of metalloproteinase; *IL*, interleukin; *TNF*, tumor necrosis factor-α; *IGF1*, insulin-like growth factor-1; *TGFB1*, transforming growth factor-β; *NGF*, nerve growth factor; *NGFR*, nerve growth factor receptor; *COL1A1*, α1 type I collagen; *COL2A1*, α1 type II collagen; *ACTA2*, actin alpha 2; *SOX9*, SRY-box transcription factor 9; *GAPDH,* glyceraldehyde-3-phosphate dehydrogenase

First, the gene expression levels among the three groups were compared without considering the site of tissue collection. In each group, the average expression levels of each gene at the three sites were calculated. As for MMPs, the expression of *MMP2* was significantly higher in the FS and RCT groups than in the SI group (*p* = 0.003 and 0.009, respectively) (Fig. 1). The expression levels of *MMP3, MMP9,* and *MMP13* were significantly higher in the FS group than in the SI and RCT groups (*p* < 0.001, *p* = 0.015 for *MMP3*, *p* < 0.001, *p* = 0.01 for *MMP9*, and both *p* < 0.001 for *MMP13*).

All three cytokines measured in this study were highly expressed in FS. The expression of *IL1B* was significantly higher in the FS group than in the SI group (*p* = 0.001). The expression levels of *IL6* and *TNF* were significantly higher in the FS group than in the SI and RCT groups (both *p* < 0.001 for *IL6*, *p* = 0.001, 0.006 for *TNF*). The expression levels of *COL1A1, COL2A1,* and *SOX9* were significantly higher in the FS group than in the SI and RCT groups (both, *p* < 0.001 for *COL1A1*, *p* = 0.01, 0.02 for *COL2A1*, and both, *p* < 0.001 for *SOX9*).

### Comparison of gene expression at three sites

**Fig. 2:**
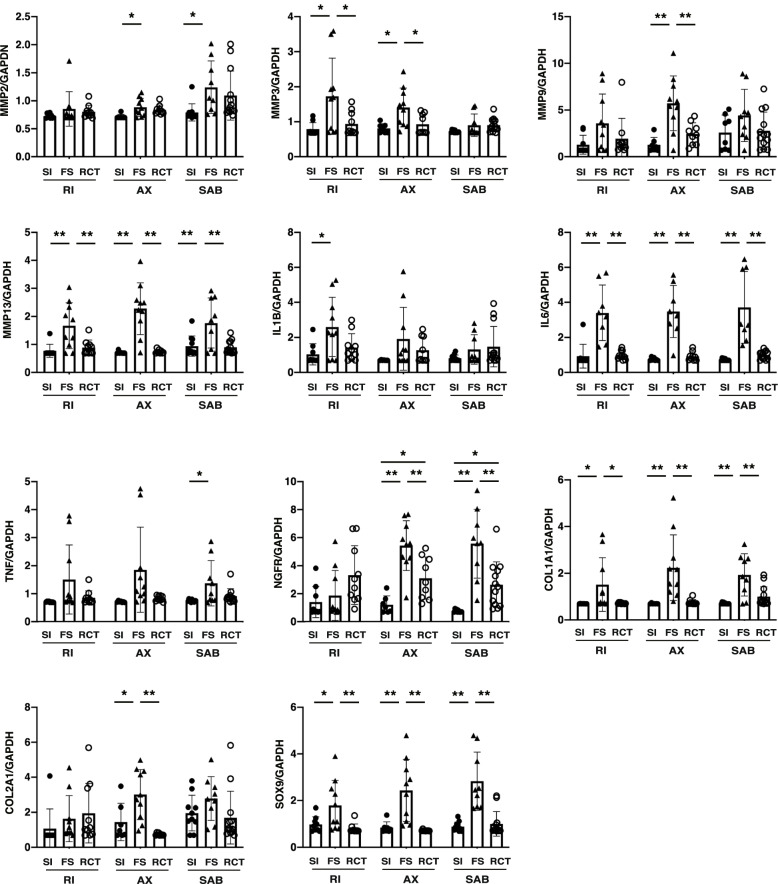
Gene expression by site. Data were presented on a logarithmic scale. *n* = 10 for each bar chart. * *p* < 0.01, ** *p* < 0.05; the Kruskal–Wallis test with Dunns multiple comparison test. Abbreviations: SI, shoulder instability; FS, frozen shoulder; RCT, rotator cuff tear; RI, rotator interval; AX, axillary recess; SAB, subacromial bursa; *MMP*, matrix metalloprotease*; IL*, interleukin; *TNF*, tumor necrosis factor-α; *IGF1*, insulin-like growth factor-1; *TGFB1*, transforming growth factor-β; *NGF*, nerve growth factor; *NGFR*, nerve growth factor receptor; *COL1A1*, α1 type I collagen; *COL2A1*, α1 type II collagen; *ACTA2*, actin alpha 2; *SOX9*, SRY-box transcription factor 9; *GAPDH,* glyceraldehyde-3-phosphate dehydrogenase

The genes that showed significant differences in Fig. 1 were further examined by site (Fig. 2). The expression levels of *MMP13, IL6, SOX9*, and *COL1A1* were higher in FS than in SI and RCT at three sites (all, *p* < 0.01 for *MMP13* and *IL6*; *p* < 0.05 for *COL1A1* in RI; *p* < 0.01 for *COL1A1* in AX and SAB; *p* < 0.05 for *SOX9* in RI; SI vs. FS, *p* < 0.01 for others in *SOX9*). Four genes (*MMP3*, *MMP9*, *COL2A1*, and *NGFR*) were increased in the AX. *MMP3* in the RI and *NGFR* in the SAB were increased in the FS group than in the RCT and SI groups (*p* < 0.05 for *MMP3* in the RI and AX; *p* < 0.01 for *MMP9* in the AX; *p* < 0.01 for *NGFR* in the AX and SAB; *p* < 0.05 for *COL2A1* in the AX; SI vs. FS, *p* < 0.05, RCT vs. FS, *p* < 0.01).

### Correlation among the expression of genes in the FS group

**Fig. 3:**
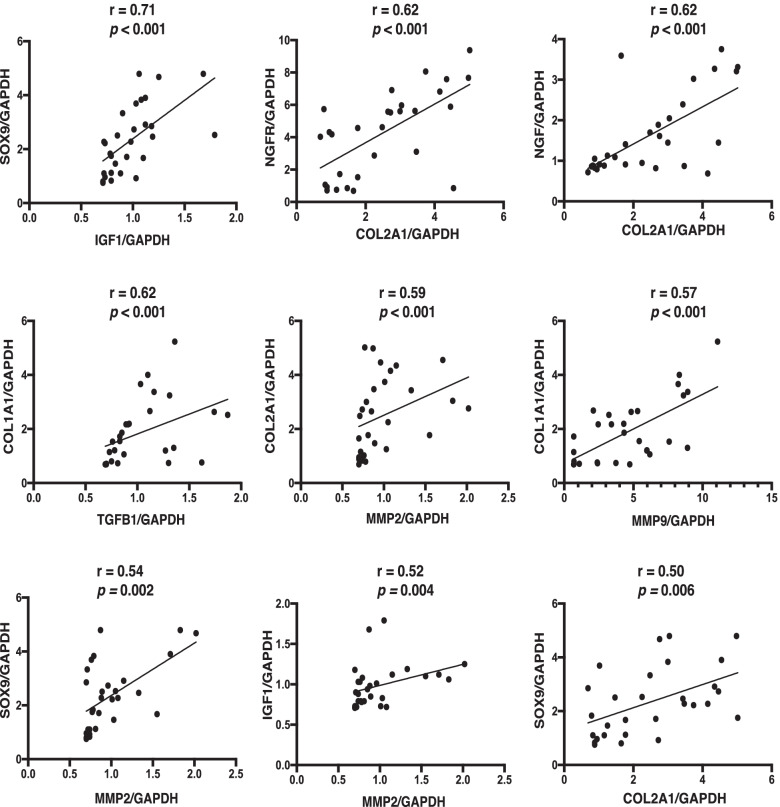
Correlation of gene expression in the FS group. Scattergrams of Spearman correlation coefficients above 0.5 are demonstrated in Table 3. Abbreviations: *SOX9*, SRY-box transcription factor 9; *IGF1*, insulin-like growth factor-1; *NGFR*, nerve growth factor receptor;* COL2A1*, a1 type II collagen;* NGF*, nerve growth factor;* COL1A1*, a1 type I collagen;* TGFB1*, transforming growth factor-b; *MMP*, matrix metalloprotease; *GAPDH, *glyceraldehyde-3-phosphate dehydrogenase

The correlation coefficients of the expression of each gene at the three sites in the FS group are shown in Table 3. Significant correlation coefficients among the expression levels of genes were found in 30 pairs. Those with correlation coefficients greater than or equal to 0.50 were indicated in Fig. 3.

**Table 3:** Results of Spearman correlation coefficients (*r*) between gene expression in the frozen shoulder group

	MMP1	MMP2	MMP3	MMP9	MMP13	TIMP1	TIMP2	TIMP3	IL1β	IL-6	TNF	IGF1	TGFB1	COL1A1	COL2A1	SOX9	NGF	NGFR
MMP2	0.04																	
MMP3	0.11	-0.33																
MMP9	0.09	0.06	0.31															
MMP13	-0.01	-0.17	-0.13	0.12														
TIMP1	-0.18	-0.29	0.09	**-0.43**	**-0.41**													
TIMP2	0.22	0.33	-0.35	-0.18	-0.10	0.00												
TIMP3	0.09	-0.21	0.18	-0.13	0.08	0.15	-0.06											
IL1β	0.14	-0.28	0.01	-0.18	0.08	-0.02	-0.06	0.35										
IL-6	-0.28	-0.13	0.03	0.21	**-0.37**	-0.01	-0.33	-0.13	0.12									
TNF	-0.35	0.36	0.13	**0.39**	0.08	-0.25	-0.24	-0.19	-0.25	0.24								
IGF1	0.34	**0.52**	0.05	0.08	-0.14	-0.39	0.25	-0.09	-0.07	-0.12	0.32							
TGFB1	0.08	0.12	0.35	**0.49**	0.09	-0.31	0.25	0.17	0.05	-0.13	0.03	0.30						
COL1A1	-0.37	0.11	0.13	**0.57**	0.34	-0.18	0.12	-0.05	-0.21	-0.02	**0.38**	-0.07	**0.62**					
COL2A1	0.17	**0.59**	-0.34	-0.22	-0.08	-0.14	0.33	-0.36	-0.11	-0.07	0.27	0.34	-0.07	-0.07				
SOX9	**0.49**	**0.54**	-0.34	-0.20	-0.11	-0.25	0.29	-0.09	0.06	-0.23	0.14	**0.71**	-0.15	**-0.40**	**0.50**			
NGF	-0.13	0.33	-0.22	-0.31	-0.05	-0.04	0.18	**-0.49**	**-0.38**	0.00	**0.43**	0.29	**-0.39**	-0.17	**0.62**	0.32		
NGFR	0.29	**0.38**	-0.36	0.06	0.01	**-0.41**	0.34	**-0.40**	0.06	0.14	0.17	0.27	0.02	0.09	**0.62**	**0.48**	0.26	
ACTA2	0.01	**0.45**	-0.21	0.26	0.04	-0.31	0.36	-0.25	-0.06	0.18	**0.48**	**0.38**	0.21	0.28	**0.45**	0.33	0.34	**0.49**

Bold values are statistically significant (*p* < 0.05)

Abbreviations: *MMP* matrix metalloprotease, *TIMP* tissue inhibitor of metalloproteinase, *IL* interleukin, *TNF* tumor necrosis factor-α, *IGF1* insulin-like growth factor-1, *TGFB1* transforming growth factor-β, *NGF* nerve growth factor, *NGFR* nerve growth factor receptor, *COL1A1* α1 type I collagen, *COL2A1* α1 type II collagen, *ACTA2* actin alpha 2, *SOX9* SRY-box transcription factor 9

## Discussion

Severe FS is often resistant to treatment and can therefore result in long-term functional impairment [7]. Despite much research, the pathogenic mechanisms related to the development of FS remain unclear. Since RI release alone did not provide a sufficient ROM, it was thought that the pathological changes affected the entire joint [6]. It was suggested that there was a difference in gene expression of tissues in FS among different sites [2, 6]. In the present study, synovial tissue was collected from three sites. We determined the characteristics of 19 gene expressions in FS and analyzed the correlation between gene expression levels.

MMPs, TIMPs, and cytokines play important roles in the pathogenesis of inflammation and fibrosis of the joint capsule in patients with FS [1]. Previous studies reported that MMP-2, -3, -9, and -13; IL-1β; TNF-α; and IL-6 were increased in the joint capsule of FS [4, 13]. Here, although gene expression mean values in the three sites demonstrated no significant differences in TIMP expression, several MMPs (*MMP3*, *MMP9*, and *MMP13)* and cytokines (*IL6* and *TNF*) were increased in the FS group than in the SI and RCT groups, suggesting a catabolic change. In contrast, *COL1A1*, *COL2A1*, and *SOX9* were increased in FS, suggesting that there was an anabolic change in the FS group. These results suggested that both catabolic and anabolic changes occurred in parallel in the FS group.

Site-specific analysis showed that *MMP13*, *IL-6*, *COL1A1*, and *SOX9* were significantly increased in all FS three sites compared to in RCT and SI, suggesting a possible involvement of these four genes in the entire joint pathogenesis of FS. The number of increased genes (FS > RCT and SI) was highest in AX (*MMP3, MMP9, NGFR,* and *COL2A1*), followed by one in SAB (*NGFR*) and one in RI (*MMP3*). Although many reports on gene expression in FS examined tissues around the RI [1], only one study examined IGHL near the AX site [6]. Hagiwara et al. reported that type III procollagen, alpha-smooth muscle actin, substance P, calcitonin, and calcitonin gene-related peptides were significantly increased in the IGHL in the FS group. Our results also demonstrated that numerous genes were upregulated in the AX. Analyzing the gene expression of the lower region of the shoulder joint might help us shed light on FS pathogenesis.

The highest correlation coefficients between the genes in the FS group were *IGF1*, *SOX9*, *COL2A1*, *NGFR*, and *NGF*. IGF-1 reportedly increased the expression of SOX9 in human articular chondrocytes [14]. NGF transgenic mice showed an increased expression of *COL2A1* and *SOX9* [15]. These reports suggested that IGF-1 and NGF induced chondrogenic differentiation. In this study, the expression levels of *IGF1*, *SOX9*, and *COL2A1* were also correlated with the *MMP2* expression. MMP-2 was reported to be a chondrogenic marker in human adipose-derived stem cells [16]. These results suggested that *MMP2* might be involved in the chondrogenesis of the FS synovium. Furthermore, the expression levels of *MMP9*, *COL1A1*, and *TGFB1* were positively correlated with each other in the FS group. Since MMP-9 was reported to induce the activation of TGF-β, which was known as a profibrotic factor [17, 18], it was suggested that *MMP9*, *COL1A1*, and *TGFB1* were involved in fibrosis. From these results, we considered that there was a high correlation between genes associated with chondrogenesis (*MMP2*, *IGF1*, *SOX9*, *COL2A1*, *NGF*, and *NGFR*) and fibrosis (*MMP9*, *TGFB1*, and *COL1A1*) in the FS group. These genes may be involved in the limited ROM of the shoulder joint in FS. Fibrotic and chondrogenic processes were reported to coexist in the articular capsule of FS and in the ligamentum flavum of lumbar spinal canal stenosis [6, 19]. The synovium contained a large number of stem cells, which had various differentiation abilities [20]. Differences in the environment within the joint might cause cells within the synovium to differentiate into fibrogenesis or chondrogenesis. Regarding the fibrotic changes in FS, several reports showed an active fibroblast proliferation with conversion to myofibroblasts [5], while others found no evidence of myofibroblast proliferation [6]. Our results showed that the mean value of *ACTA2* expression, a marker of myofibroblasts, did not increase in the FS group.

One of the symptoms of FS is pain [21]. NGFR is a neural marker, and *NGFR* was found to be increased in the AX and SAB in the FS group. In FS, previous arthroscopic and imaging findings have revealed changes in the RI [1], but the results of our study showed no changes in *NGFR* in the RI. Molecules other than NGFR have also been reported to be involved in FS pain. Xu et al. reported increased levels of some neuronal proteins in FS, including growth-associated protein-43, protein gene product 9.5, and CD34 [11]. Further research is required to elucidate the molecular mechanisms governing FS pain.

Our study has several limitations. First, mRNA expression was evaluated, while protein synthesis was not determined. Second, the sample size of this study was small. However, compared to previous similar studies, our sample size was comparable. Third, many patients with FS were often cured conservatively, so it was possible that we were collecting non-normal FS cases. Fourth, the age difference between the three groups was not equal, which was one of the limiting factors. Fifth, the effectiveness of steroid injections has not been evaluated. Since most of the patients went to other clinics before visiting the enrolled hospitals, we do not know if they received steroid injections. All patients did not receive steroid injections in the three weeks between their initial visit to the hospital and their surgery.

## Conclusion

The expression levels of numerous *MMPs*, pro-inflammatory cytokines, and collagen-related genes were increased in the FS group, suggesting that catabolic and anabolic changes occurred in parallel. In addition, genes related to chondrogenesis or fibrosis were highly expressed in the FS group, which might affect the shoulder′s ROM in the shoulder. Compared to RI and SAB, AX was the most common site of increased expression in FS. The analysis of the lower region of the shoulder joint may lead to the elucidation of the pathogenesis of FS.

## Data Availability

The datasets analyzed during the current study are available from the corresponding author on reasonable request.

## Abbreviations

FS: Frozen shoulder
RCT: Rotator cuff tear
SI: Shoulder instability
RI: Rotator interval
AX: Axillary recess
SAB: Subacromial bursa
MMPs: Matrix metalloproteinases
TIMPs: Tissue inhibitors of metalloproteinases
NGF: Nerve growth factor
NGFR: Nerve growth factor receptor
MRI: Magnetic resonance imaging
MGHL: Middle glenohumeral ligament
IGHL: Inferior glenohumeral ligament
ROM: Range of motion
cDNA: Complementary DNA
IL: Interleukin
TNF: Tumor necrosis factor
IGF-1: Insulin-like growth factor-1
TGFB1: Transforming growth factor-β
COL1A1: α1 Type I collagen
COL2A1: α1 Type II collagen
ACTA2: α-Smooth muscle actin
SOX9: SRY-box transcription factor 9
GAPDH: Glyceraldehyde-3-phosphate dehydrogenase
